# The Allometry of Bee Proboscis Length and Its Uses in Ecology

**DOI:** 10.1371/journal.pone.0151482

**Published:** 2016-03-17

**Authors:** Daniel P. Cariveau, Geetha K. Nayak, Ignasi Bartomeus, Joseph Zientek, John S. Ascher, Jason Gibbs, Rachael Winfree

**Affiliations:** 1 Department of Entomology, University of Minnesota, Saint Paul, Minnesota, United States of America; 2 Department of Ecology, Evolution, and Natural Resources, Rutgers University, New Brunswick, New Jersey, United States of America; 3 Departamento Ecología Integrativa, Estación Biológica de Doñana (EDB-CSIC), Avda. Américo Vespucio s/n, Isla de la Cartuja, 41092, Sevilla, Spain; 4 Department of Biological Sciences, National University of Singapore, 14 Science Drive 4, Singapore, 117543, Singapore; 5 Department of Entomology, Michigan State University, East Lansing, Michigan, United States of America; University of North Carolina, Greensboro, UNITED STATES

## Abstract

Allometric relationships among morphological traits underlie important patterns in ecology. These relationships are often phylogenetically shared; thus quantifying allometric relationships may allow for estimating difficult-to-measure traits across species. One such trait, proboscis length in bees, is assumed to be important in structuring bee communities and plant-pollinator networks. However, it is difficult to measure and thus rarely included in ecological analyses. We measured intertegular distance (as a measure of body size) and proboscis length (glossa and prementum, both individually and combined) of 786 individual bees of 100 species across 5 of the 7 extant bee families (Hymenoptera: Apoidea: Anthophila). Using linear models and model selection, we determined which parameters provided the best estimate of proboscis length. We then used coefficients to estimate the relationship between intertegular distance and proboscis length, while also considering family. Using allometric equations with an estimation for a scaling coefficient between intertegular distance and proboscis length and coefficients for each family, we explain 91% of the variance in species-level means for bee proboscis length among bee species. However, within species, individual-level intertegular distance was a poor predictor of individual proboscis length. To make our findings easy to use, we created an R package that allows estimation of proboscis length for individual bee species by inputting only family and intertegular distance. The R package also calculates foraging distance and body mass based on previously published equations. Thus by considering both taxonomy and intertegular distance we enable accurate estimation of an ecologically and evolutionarily important trait.

## Introduction

Allometric relationships underlie many important patterns in ecology and evolution. Body size has been shown to scale with metabolic rate and this may in turn explain variation in life history traits, species interactions and the rate of ecological processes [1]. Allometric relationships can reveal interesting patterns in life history evolution [2–4]. For example in mammals, the allometric relationship between body size and growth rate differs among species based on life history traits such dietary specialization and death rate [2]. Because allometric relationships are often phylogenetically conserved, knowledge of allometric relationships can be used to identify taxa that diverge from the expected pattern. These taxa often have atypical life histories or exaggerated traits that may be the result of unique patterns of selection [5, 6]. Within community ecology, allometric relationships can underlie key traits involved in resource acquisition, and are used to predict competitive interactions [7].

The predictive nature of allometric relationships makes them a potentially useful tool for estimating ecologically important traits that are otherwise difficult to measure. Here we take this approach to develop a predictive allometric equation for proboscis length in bees (Hymenoptera: Apoidea: Anthophila). Proboscis length mediates multiple aspects of bee ecology and evolution including flower choice [8, 9], efficiency in acquiring floral resources [10–12], pollination effectiveness [13, 14], community assembly ([15], but see [16]), the structure of plant-pollinator networks [17], and possibly even plant speciation [14, 18, 19]. Proboscis length may predict which bee species undergo population declines due to global change [20, 21], and which species benefit most from pollinator restoration programs [22]. However, few studies have examined proboscis length outside of the genus *Bombus*, thus limiting its applicability in community ecology and macroecology (but see [17]).

Proboscis length in bees can be difficult to measure, requiring dissection of the mouthparts (glossa and prementum), ideally in fresh specimens, which must first be identified to the species level because dissection may damage specimens. Measurement of proboscis length is particularly difficult for small bees. For these reasons, the few studies that have measured proboscis length for multiple bee species have focused on large-bodied species, such as bumble bees (*Bombus*), because their proboscis length is relatively easy to measure (e.g. [16, 20, 23] but see [17]). A common practice in pollination ecology is to use family or other phylogenetic groupings as a proxy for proboscis length [24–26]. In particular, the sister families Megachilidae and Apidae comprise the long-tongued families [27], a monophyletic group diagnosed in part by homologous modifications of the proboscis, notably elongate and flattened labial palpi and an elongate glossa [28]. These are contrasted with short-tongued bees, comprising all other bee families, which are considered a paraphyletic assemblage, although the nature of this paraphyly is controversial due to conflict between and among morphological and molecular phylogenetic studies [29, 30]. Bumble bees (genus *Bombus*) are likewise often divided into subgenera that have long and short proboscises [31], among which the phylogenetic relationships have recently been resolved through molecular phylogenetic analyses [32, 33]. While it is clear that proboscis length varies in general among bees, a central interest in many studies is to infer absolute or functional length of the proboscis to address ecological hypotheses that are related to the ability of pollinators to access floral rewards. Further, there is tremendous variation in functional length with these groups. However, a quantification of the variation in functional length of bee tongues has not been done outside of *Bombus*. Most importantly, allometric scaling of functional tongue length may be modified by phylogenetic relationships, but this has not yet been explored for pollinators.

In this study, we develop a predictive equation for estimating bee proboscis length as a function of family (taxonomy) and body size (allometry). We fit the equation and estimate coefficients based on almost 800 empirical measurements of proboscis length from 100 bee species of 5 families. We provide an R package that allows users to input intertegular span and family to estimate proboscis length for any species of bee that belongs to five of the seven extant bee families. These five families include 99% of the world's bee species.

## Material and Methods

### Data collection and morphological measurements

To measure proboscis length on a diverse set of native, wild bees, we collected bees at 35 sites throughout the state of New Jersey, USA from 2012 to 2014. Specimens were collected on private property with permission from private land owners. All measurements were done on fresh specimens, using a Nikon SMZ800 stereoscope. To measure proboscis length and body size, we used Nikon NIS-Elements software (Ver. 3.07).

**Fig 1:**
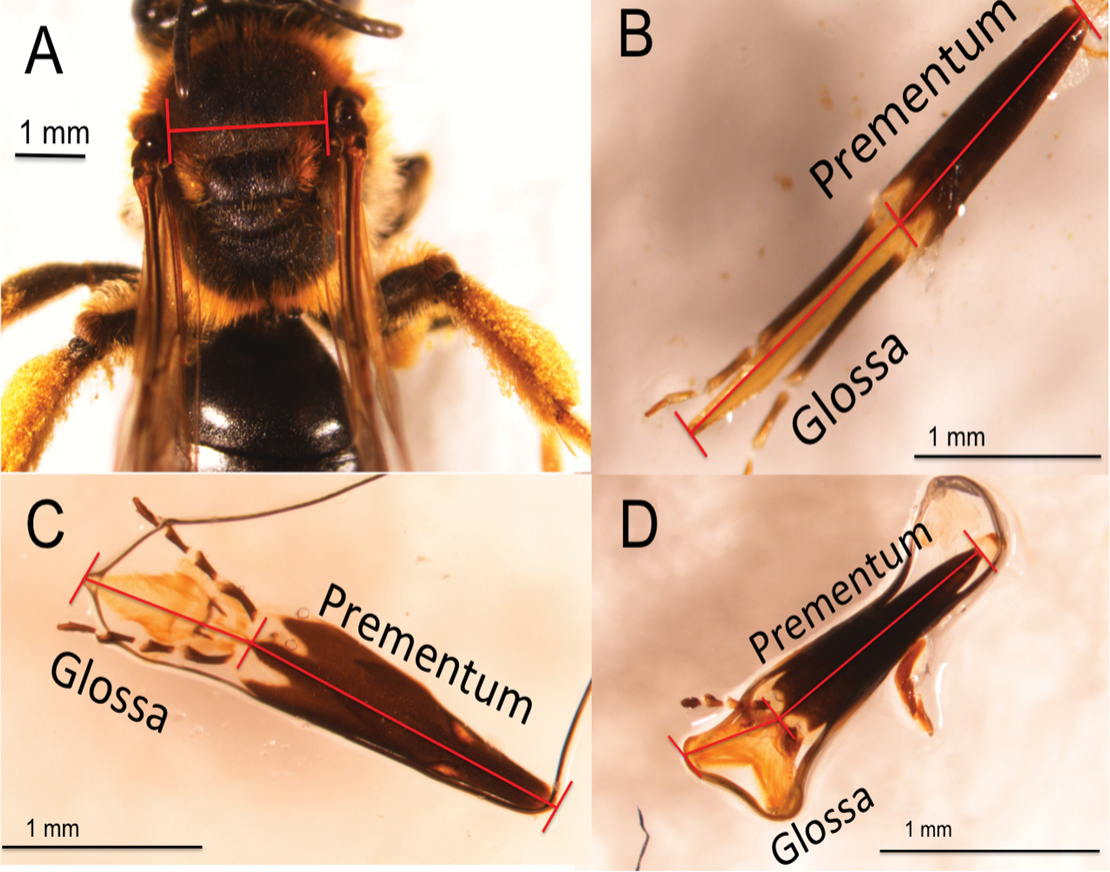
Photographs of intertegular distance (IT), glossa, prementum and proboscis. (A) Photographs of IT for *Andrena wilkella* Kirby (Andrenidae) (B) Glossa, prementum and proboscis length in the long-tongued bee *Nomada illinoensis* Robertson (Apidae), and two short-tongued bees (C) *Andrena bradleyi* Viereck (Andrenidae) a long-faced Andrena, and (D) *Colletes inaequalis* Say (Colletidae).

We measured proboscis length as follows. The *prementum* was measured as the distance from the base of the mentum to the distal point of the basiglossal sclerite ([27], Fig 1B–1D). The *glossa* was measured from the basiglossal sclerite to the distal point of the labellum as in Harder 1982 ([23] Fig 1B–1D). It was common for glossae to be retracted in or folded along the prementum. In these cases, special care was taken to remove or extend the glossa. All measurements were conducted only after glossae were completely extended from the prementum [23]. We defined *proboscis length* as the length of the glossa plus the prementum.

We measured bee body size as the distance between the tegulae, or wing bases (*intertegular distance*; hereafter, *IT*, Fig 1A). This is the standard measure of body size in bees as it is highly correlated with dry body mass ([34], R2 = 0.97, N = 20) which is itself problematic to measure particularly for pinned specimens. Ecological studies of bees have used IT to predict flight distance [35] and other outcomes related to organism mobility such as response to land cover at various scales [36, 37].

### Data analysis

To examine the *interspecific* relationship between intertegular distance, taxonomy and proboscis length, we used a power function, as is commonly done in allometric studies [23, 38]:

ln(Y)=ln(a)+b*ln(IT) (Equation 1)

Where Y = length of proboscis, glossa, or prementum in mm, a = a coefficient specific to the bee family, IT = intertegular distance (IT) in mm, and b = the allometric scaling coefficient. While overall proboscis length was the primary response variable of interest, the prementum and glossa individually may also be important for bee foraging [11]. Therefore we also used this equation separately for analysis of prementum and glossa lengths. The family-specific coefficient (*a*) allows for different intercepts for each family. The scaling constant (*b*) reflects the slope of the relationship between IT and proboscis length. Due to sample size limitations for some of the rarer species, we were limited in our ability to test for differences between sexes. However, analyses run with only females (which constitute the majority of individuals collected in most studies) versus with both females and males yielded similar results; thus we combined the sexes in further analyses.

We then used model selection to determine which predictor variables provided the best-fitting model based on lowest AIC value. The presence of the family coefficient (a) in the best-fitting model indicates that intercepts differ among families. The presence of the scaling coefficient (b) in the best-fitting model indicates that proboscis, glossa or prementum scales with intertegular distance. An interaction between family and IT indicates that the scaling coefficient (b) differs among families. In addition, to determine whether simply long- or short-tongued grouping predicted proboscis length, we also conducted model selection using IT, tongue-type (short- versus long-tongued bees), and their interaction. The model that includes only tongue-type is equal to assumptions made in pollination studies that use this category without considering allometry. We used values from the best fitting model as parameter values for Eq 1.

In addition to OLS regression that is best suited to our predictive purposes [39], we also used standardized major axis estimation (SMA) to test whether the family coefficients (a) and scaling coefficients (b) differed among models. This analysis is commonly used in allometry studies to summarize the relationships among variables as it incorporates error in the X variable (in our case IT) as well as the Y variable [39]. For the glossa, prementum and proboscis, we tested for differences in slopes and intercepts among families using the *sma* function in the *smatr* package in R [40]. As SMA approaches are highly sensitive to outliers, we used the robust SMA estimation [40, 41].

To determine whether this allometric relationship could explain within-species variation in proboscis length for bees, we also ran a set of *intraspecific models*, one for each bee species that had 10 or more individuals measured. Individual-level IT was the predictor. Because males, females, and in social bees, queens, can vary in morphology, we included sex or caste as a predictor for those species for which we had representatives from each sex or caste.

Lastly, we tested the generality of the model we developed above for bees, using data on butterfly body size and proboscis length as reported by Kunte [7]. These data include species-level means for body length (as a proxy for body size) and proboscis length for 117 species of butterflies in 6 different families in Costa Rica. We conducted this step for the interspecific model only because the butterfly data set contained only species-level means for body length and proboscis length.

## Results

We measured 786 specimens of 100 species belonging to 28 genera of 5 of the 7 extant bee families. We lacked data for two families: Melittidae and Stenotritidae. Melittidae is limited to one relatively common species in our region, with the others being rarely collected. Therefore we were unable to predict values across Melittidae. Stenotritidae are restricted to Australia. Species-level means for IT, glossa, prementum, and proboscis are included as supplementary information (S1 Table).

**Fig 2:**
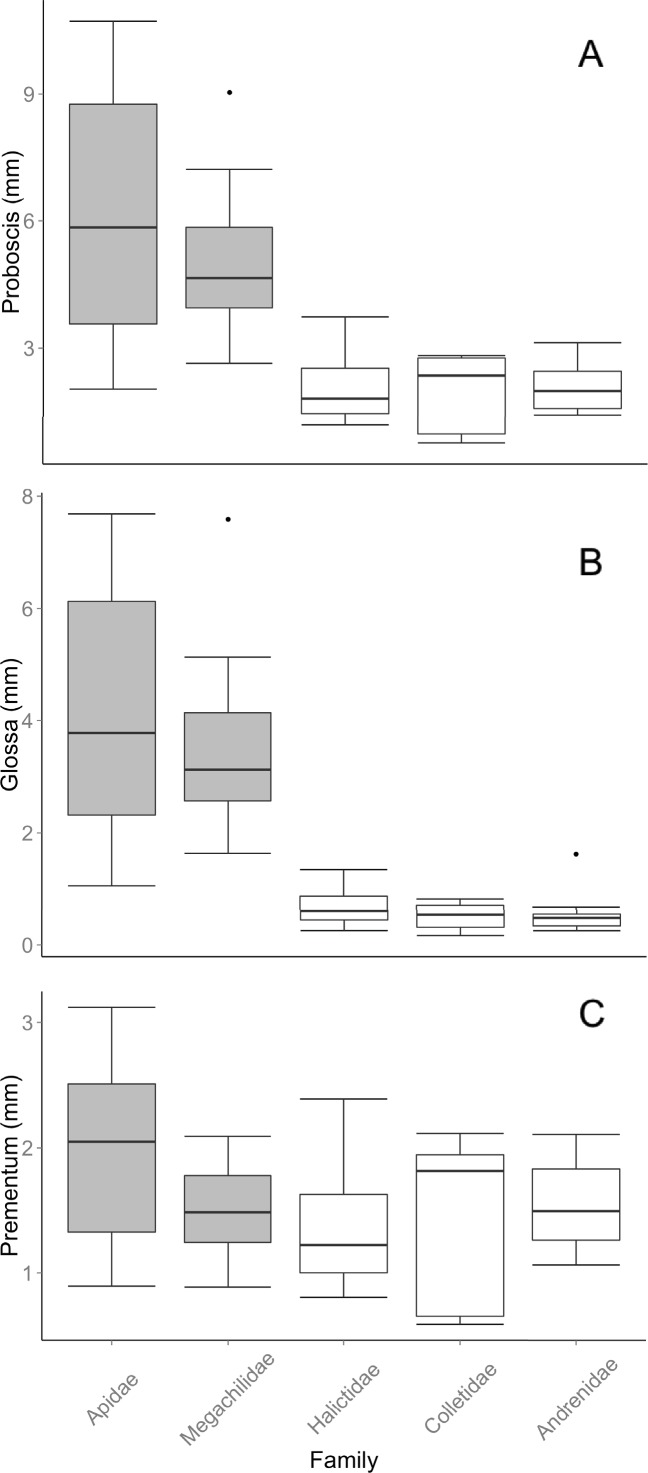
Proboscis, glossa, and prementum length. Boxplots of (A) proboscis, (B) glossa, and (C) prementum length for the five families of bees measured. Grey boxplots are long-tongued families while clear boxplots represent short-tongued families. Dots represent outliers. Figures are drawn using raw data.

In the interspecific models using OLS regression, the combination of family and mean IT strongly predicted the mean length of the proboscis, glossa and prementum. Family and IT were retained in the best model selected by AIC in all cases (Table 1), and model R^2^ values were 0.91, 0.85, 0.91 for glossa, prementum and proboscis, respectively. Including the interaction between family and IT did not provide a better fit for the proboscis or glossa model while the interaction improved the fit of the prementum model (Table 1). The robust SMA analysis obtained results qualitatively similar to those found by OLS regression. We found no evidence for an interaction between family and IT for either the glossa or proboscis model (glossa: likelihood ratio = 7.3, df = 4, *P* = 0.12; proboscis: likelihood ratio = 4.5, df = 4, *P* = 0.34). There was evidence for an interaction between family and IT in the prementum SMA model (likelihood ratio = 15.3, df = 4, *P* = 0.004). In all the OLS regression models, family was retained in the best-fit models indicating that mean length of mouthparts differed among families (Table 1). Similarly, in all three SMA models, intercepts differed among families (glossa: *W*_c_ = 465.1, df = 4, *P* = <0.001; prementum: *W*_c_ = 465.1, df = 4, *P* = <0.001l proboscis: *W*_c_ = 465.1, df = 4, *P* = <0.001). In general, long- and short-tongued families differed in proboscis and glossa length, with the two long-tongued families having the longest proboscises and glossae, although prementum length was more similar (Table 2, Fig 2). The R^2^ for the OLS regression models were lower for family alone (glossa = 0.53, prementum = 0.69, proboscis = 0.73, Table 1) or only long vs. short-tongued family groups (glossa = 0.77, prementum = 0.08, proboscis = 0.61, Table 1), or for IT alone (glossa = 0.79, prementum = 0.16, proboscis = 0.62, Table 1).

**Table 1:** AIC and R^2^ values for interspecific models. Models are presented in ascending order of AIC value. Top models used for estimates are in bold. Models with + do not include an interaction term, whereas models with x do include the interaction.

Response Variable	Model	R^2^	AIC
Proboscis	**Family + IT**	**0.91**	**-41.91**
	Family x IT	0.91	-38.96
	Short- vs. Long-Tongued + IT	0.87	-11.1
	Short- vs. Long-Tongued x IT	0.87	-10.6
	IT Only	0.73	61.7
	Short- vs. Long- Tongued Only	0.61	99.06
	Family Only	0.62	102.6
Glossa	**Family + IT**	**0.91**	**67.43**
	Family x IT	0.91	71.06
	Short- vs. Long-Tongued x IT	0.87	98.7
	Short- vs. Long-Tongued + IT	0.86	102.2
	Family Only	0.79	149.7
	Short- vs. Long-Tongued Only	0.77	151.14
	IT Only	0.53	222.9
Prementum	**Family x IT**	**0.85**	**-100.11**
	Family + IT	0.82	-92.1
	Short- vs. Long-Tongued x IT	0.74	-56.2
	Short- vs. Long-Tongued + IT	0.75	-55.9
	IT Only	0.69	-41.9
	Family Only	0.16	66.9
	Short- vs. Long-Tongued Only	0.08	69.53

Using the butterfly data from Kunte (2007), we found that the combination of family and body length (AIC = 24.3 with a body length by family interaction term) compared favorably to either family (AIC = 105.8) or body length (AIC = 101.8) alone. The R^2^ values were also higher when combining body length and family (R^2^ = 0.77 for both factors combined, versus R^2^ = 0.47 for body length and R^2^ = 0.25 for family).

**Fig 3:**
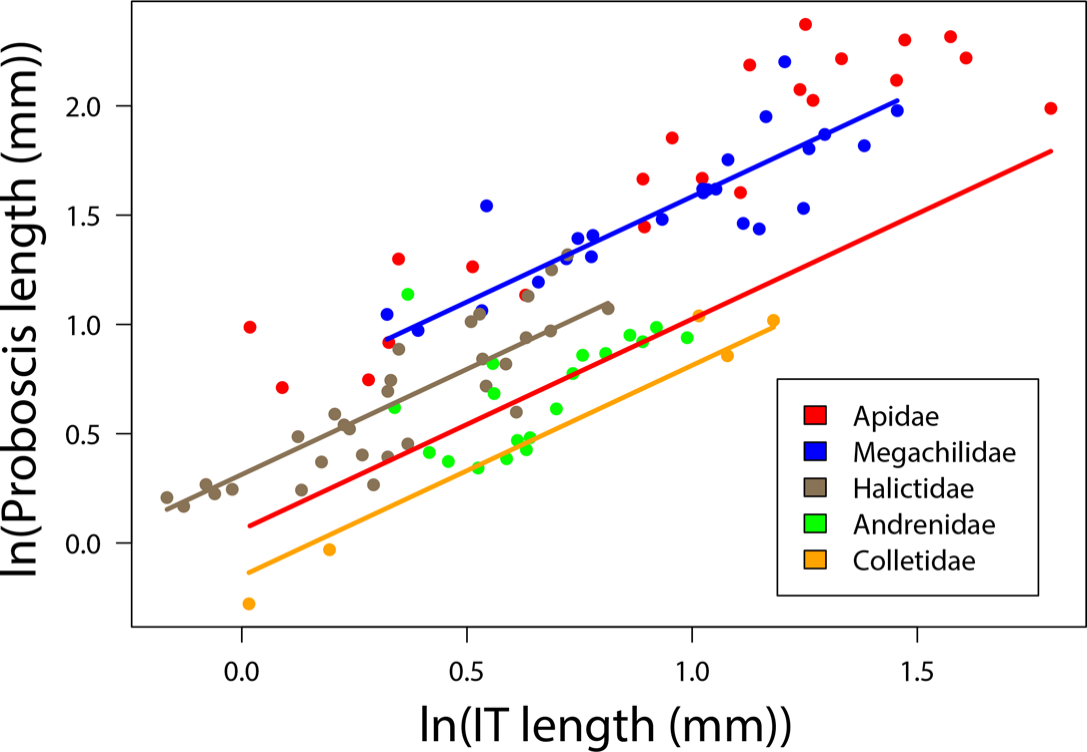
Relationship between IT and proboscis length. The relationship between intertegular distance (IT) and proboscis length in 101 species of bees. Each point represents the mean IT and proboscis length for a bee species. Colors are bee families. Lines are fit using regression coefficients from model outputs. Both IT and proboscis length are ln transformed.

We used the estimates of the OLS regression models with the lowest AIC value for the parameter values in the allometric power function (Eq 1). As the slopes do not differ among families for the proboscis and glossa models (no interaction between IT and family, Table 1), the value for b is the same across families. The interaction was retained in the best model for prementum and therefore, each family has a specific IT scaling constant. Model-estimated values for a and b are in Table 2. Fig 3 shows the family-specific allometric relationship between IT and proboscis length. The IT coefficient was nearly 1 and thus indicates a linear relationship between IT and proboscis length (Table 2).

**Table 2:** Estimates for the coefficients a and b for fitting Eq 1, log(*Y*) = log(*a*) + log(*I*) * *b*, where Y = length of proboscis, glossa, or prementum in mm, a = a coefficient specific to the bee family, I = intertegular distance (IT) in mm, and b = the allometric scaling constant, and logs are base e. Coefficients are from the interspecific OLS regression models with the lowest AIC values (Table 1).

Response Variable	Family	Family Coefficient (*a*)	IT Coefficient (*b*)
Proboscis	Andrenidae	1.06	
	Apidae	2.13	
	Colletidae	0.86	
	Halictidae	1.38	
	Megachilidae	1.87	
		——	0.96
Prementum	Andrenidae	0.88	0.83
	Apidae	0.91	0.73
	Colletidae	0.56	1.14
	Halictidae	0.89	1.04
	Megachilidae	0.77	0.68
Glossa	Andrenidae	0.23	
	Apidae	1.27	
	Colletidae	0.21	
	Halictidae	0.43	
	Megachilidae	1.16	
		——	1.04

We focused on the relationship between IT and proboscis length for practical reasons, because it is easy to measure this trait in live and preserved bees. However, body size is a standard measure in the allometry literature so we investigated the body mass–proboscis length relationship as well. IT itself is known to have a negative allometric relationship with dry body mass such that IT does not increase as fast, in a proportional sense, with body mass (34). For this part of the paper, we used an additional analysis, SMA, which is best used to test for positive or negative allometry. SMA analyses for all mouthparts indicated a negative allometric relationship with estimated body mass, with the exception of glossa in Andrenidae in which the scaling coefficient (b) of one was within the 95% confidence interval (S2 Table). Thus overall proboscis length did not increase as quickly as body mass.

We also provide an R package, *BeeIT*, that estimates proboscis, glossa and prementum length based on IT and family for most bee species in the five families for which we have data. The R package can be downloaded at https://github.com/ibartomeus/BeeIT. These five families make up approximately 99% of all bee species, allowing for general use of this estimation. However, it is important to note that some groups such as euglossine bees, are widely known to have extremely long tongues [18]. These groups will likely have higher intercepts than other species in Apidae. The R package, *BeeIT*, also uses IT to estimate body mass ([34] S1 Fig) and typical foraging distance ([35] Table 1) using previously published equations [34, 35]. We included these equations and coefficients from the OLS regression models in the R package, *BeeIT*, as many users may wish to calculate all three quantities simultaneously.

In contrast to the interspecific models above, we found little evidence in the intraspecific models that IT strongly predicted length of mouthparts across the individuals of a species. For the 24 species analyzed, we did find that the relationship was statistically significant in 10 (glossa), 16 (prementum), and 16 (proboscis) cases. However, R^2^ values were low for most species with mean values being 0.14, 0.39 and 0.36 for glossa, prementum and proboscis respectively (S3 Table, S2 Fig). Mean R^2^ values were low even for species with a statistically significant relationship between IT and length of mouthparts (*P <* 0.05; glossa = 0.36; prementum = 0.53, proboscis = 0.50).

## Discussion

We show that proboscis length in bees, a trait that is ecologically important but difficult to measure, can be predicted with an R^2^ of 0.91 using an allometric equation that requires only information on family and IT (Figs 1 and 3). Our use of body size allometry represents a dramatic improvement over existing methods of estimating pollinator proboscis length, which used only taxonomic information. Ecologists and evolutionary biologists interested in functional proboscis length in bees have long relied on the long- vs. short-tongued family categories (e.g. [21, 24, 26]) without considering body size allometry; but this method explained only 61% of the variance in proboscis length in our large data set. The equations and coefficients we present here will increase the precision and quality of the many studies investigating the important of functional proboscis length ecology of native bee foraging behavior, resource competition, or mutualist partner choice, as well as evolutionary studies of plant-pollinator mutualisms. Our approach might generalize well to other pollinator taxa, based on our test with a data set on tropical butterflies, in which the combination of family and body size explained 77% of the variance in proboscis length, an improvement over the 47% explained by family alone.

Our estimation tool should be useful for pollinator community ecology because proboscis length is such an important trait for structuring pollinator communities and plant-pollinator networks. For example, at a single study site Stang and others [42] measured proboscis length across multiple insect pollinator taxa and found trait matching between proboscis length and corolla length of flowers visited by those pollinators. Proboscis length and its matching with corolla length is likewise an important factor structuring plant-pollinator networks involving hummingbirds [43, 44] and Lepidoptera [45]. A frequent use of proboscis length in the pollination ecology literature is to measure floral specialization with respect to nectar gathering; that is, pollinator species with long tongues are assumed to specialize on flowers with long corollas [20, 26]. However, measuring proboscis length is often not feasible for all species in a community. Our allometric equation will make these proboscis estimates more accurate and facilitate stronger community analyses. For instance, in our data set the smallest long-tongued bee species weighs approximately 2 mg while the largest is 162 mg, which leads to a nearly 6-fold empirical difference in estimated proboscis length (2.1 versus 12.1 mm). If studies examining the functional importance of proboscis length did not consider the scaling of proboscis length with body size, these studies would consider these species to have equivalent proboscis length.

The relationship we present here will also be useful in exploring why particular species and genera deviate from the predicted relationship. Deviations from allometric relationships have revealed interesting patterns in other insects. For example in Odonata, species with relatively longer wings for a given body length were migratory species [46]. In ants, controlling for body size, longer legs of some desert genera reduce foraging time and increase cooling [47]. In bees, proboscis lengths that diverge from the predicted relationship, such as those that are longer than predicted given their body size, may indicate selection pressures for flower specialization or other life history traits. For example, of the more than 1500 or more species of *Andrena* have short proboscises. Two long-tongued species, the sister species *Andrena* (Stenomelissa) *lonicera* Warncke and *A*. (S.) *halictoides* Smith are nectar specialists and likely evolved long proboscises from a common ancestor and now exploit the long corollas of their host flowers, *Lonicera gracilipes* Miq. (Caprifoliaceae) and *Weigela hortensis* (Siebold. & Zucc.) K. Koch. (Caprifoliaceae), respectively [14]. Similar patterns are found in other bee taxa and insect orders [48]. As some species and genera may deviate from the predicted allometric relationship, the equation we present here should not substitute for detailed measurements when warranted and practical.

We found differences among the glossa, prementum, and overall proboscis length. In particular, family alone explained much more variance in glossa length than in prementum length. This indicates that the result for the differences in proboscis length among families are driven primarily by glossa length. Estimating length of different mouthparts may be warranted depending on which families of bees are being studied. For example, some species of short-tongued bees extend the basal segments of their mouthparts including the prementum during feeding [11, 14]. In contrast, long-tongued bees such as *Bombus* may keep their prementum static and thus use it very little during feeding, instead extending their glossae [23].

In contrast to the strong predictive relationship that we found at the species level, we did not find strong evidence the IT consistently predicts the variation in proboscis length among individuals of the same species. This differs from earlier work showing that IT or other easy-to-measure morphological characters such as wing size and length of the radial cell of the wing showed strong relationships with proboscis length (R^2^ > 0.90), at least within *Bombus* species [9, 23, 49, 50]. Our results may differ from these previous studies as they often used specimens from single sampling locations ([9, 23, 49] but see [50]) or even a single colony [23]. Therefore, groups of closely related individuals may have more similar relationships between body size and proboscis length. In contrast, our study included bees collected across a wide geographic area, and allometric relationships can vary geographically and within species [51, 52].

Proboscis length is an important trait affecting plant-pollinator interactions but has not been routinely included as a variable in pollinator studies due to the difficulty of measuring these mouthparts, particularly in small, short-tongued bees, and it may conflict with other research goals such as specimen identification. The equation and coefficients presented here and in the accompanying R package (https://github.com/ibartomeus/BeeIT) will facilitate rapid estimation of proboscis length using only family information plus a simple body size measurement.

## Supporting Information

S1 FigThe relationship between body mass and proboscis length in 101 species of bees.Body mass is estimated using equation: in Cane 1987, Fig 1 where $\text{Body Mass}={\left(\frac{IT}{0.77}\right)}^{\frac{1}{0.405}}$. Each point represents the mean dry body mass and mean proboscis length for a bee species.(EPS)

S2 Fig Scatterplots of intraspecific relationships between IT and proboscis length. Species with 10 or more individuals are represented.
(EPS)

S1 Table Table of species-level means for IT, glossa, prementum and proboscis length. (XLSX)

S2 Table Table of family-level slopes and lower and upper 95% confidence intervals from robust SMA. (CSV)

S3 Table Table of intraspecific R^2^ values for OLS regression models of IT as a function of glossa, prementum and proboscis length. Predictor variables were IT and sex. Response variables were either glossa, prementum and proboscis length.
(XLSX)
