# Correction: The Allometry of Bee Proboscis Length and Its Uses in Ecology

**DOI:** 10.1371/journal.pone.0207900

**Published:** 2018-11-19

**Authors:** Daniel P. Cariveau, Geetha K. Nayak, Ignasi Bartomeus, Joseph Zientek, John S. Ascher, Jason Gibbs, Rachael Winfree

In Fig 3 the calculated Apidae slope is incorrect and covers the Andrenidae slope. Please see the correct Fig 3 here.

**Fig 3:**
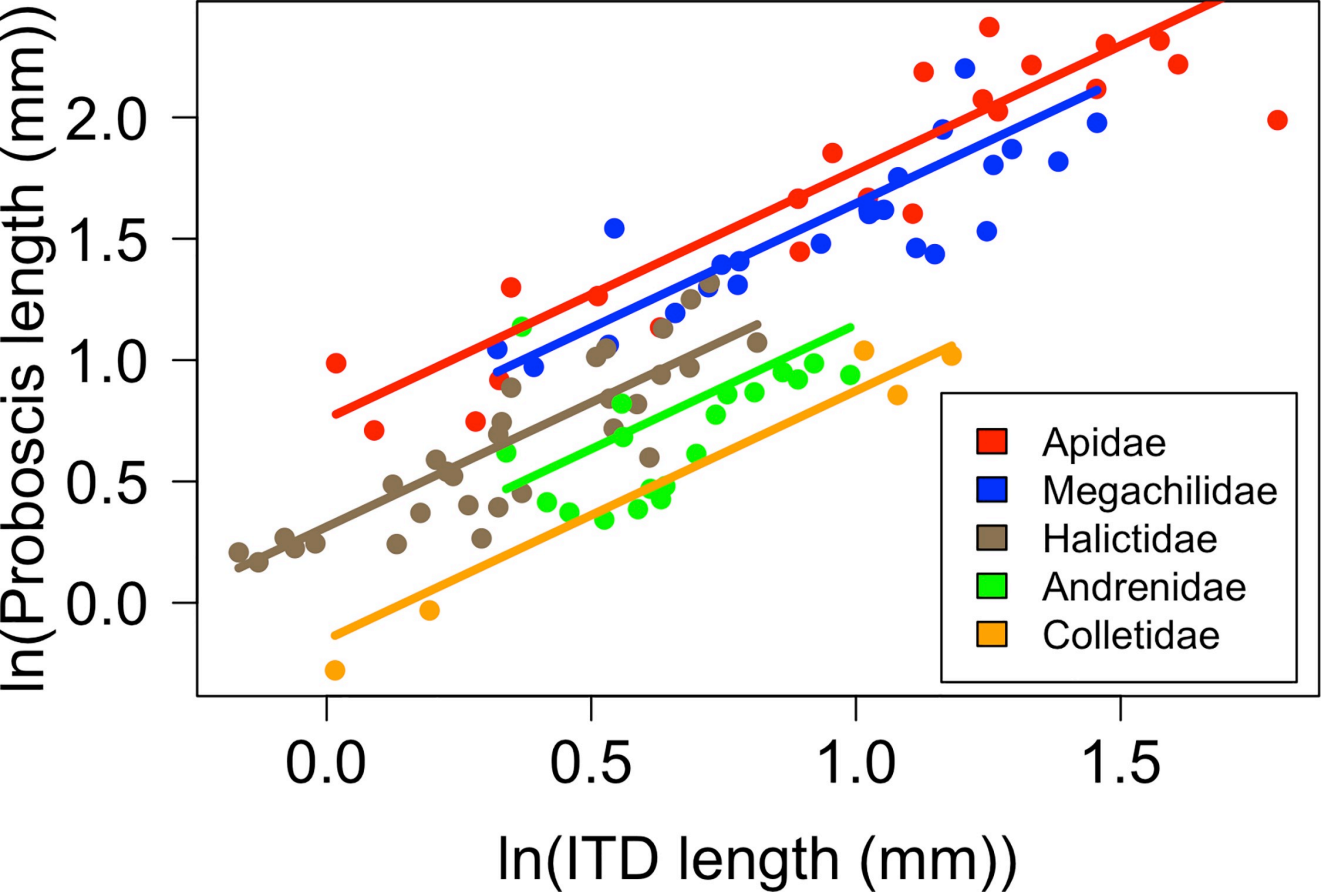
Relationship between IT and proboscis length. The relationship between intertegular distance (IT) and proboscis length in 101 species of bees. Each point represents the mean IT and proboscis length for a bee species. Colors are bee families. Lines are fit using regression coefficients from model outputs. Both IT and proboscis length are ln transformed.

## References

[1] Cariveau DP, Nayak GK, Bartomeus I, Zientek J, Ascher JS, Gibbs J, et al (2016) The Allometry of Bee Proboscis Length and Its Uses in Ecology. PLoS ONE 11(3): e0151482 10.1371/journal.pone.0151482 26986000 PMC4795761

